# An updated global atmospheric paleo‐reanalysis covering the last 400 years

**DOI:** 10.1002/gdj3.121

**Published:** 2021-05-04

**Authors:** Veronika Valler, Jörg Franke, Yuri Brugnara, Stefan Brönnimann

**Affiliations:** ^1^ Oeschger Centre for Climate Change Research University of Bern Bern Switzerland; ^2^ Institute of Geography University of Bern Bern Switzerland

**Keywords:** climate reconstruction, ensemble Kalman fitting, paleoclimate data assimilation

## Abstract

Data assimilation techniques are becoming increasingly popular for climate reconstruction. They benefit from estimating past climate states from both observation information and from model simulations. The first monthly global paleo‐reanalysis (EKF400) was generated over the 1600 and 2005 time period, and it provides estimates of several atmospheric fields. Here we present a new, considerably improved version of EKF400 (EKF400v2). EKF400v2 uses atmospheric‐only general circulation model simulations with a greatly extended observational network of early instrumental temperature and pressure data, documentary evidences and tree‐ring width and density proxy records. Furthermore, new observation types such as monthly precipitation amounts, number of wet days and coral proxy records were also included in the assimilation. In the version 2 system, the assimilation process has undergone methodological improvements such as the background‐error covariance matrix is estimated with a blending technique of a time‐dependent and a climatological covariance matrices. In general, the applied modifications resulted in enhanced reconstruction skill compared to version 1, especially in precipitation, sea‐level pressure and other variables beside the mostly assimilated temperature data, which already had high quality in the previous version. Additionally, two case studies are presented to demonstrate the applicability of EKF400v2 to analyse past climate variations and extreme events, as well as to investigate large‐scale climate dynamics.

## Graphical abstract

This paper presents the EKF400v2 global monthly paleo‐reanalysis, generated with a paleoclimate data assimilation method, covering the last 400 years. The paleo‐reanalysis provides several climate fields such as temperature, precipitation and sea‐level pressure. We compare EKF400v2 to a previous version, describe the extended observational network, the model simulations and highlight the applied modifications to the assimilation system. The paleo‐reanalysis is evaluated in the 20th century, and two case studies demonstrate the usage of the dataset to investigate past climate variability.

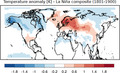

## 1 INTRODUCTION

Climate variability can be examined over several temporal and spatial timescales. To study climate variation at decadal to centennial timescales, long time series are required. Previous climate reconstructions suggest that strong natural variability characterized the period before 1850, but the underlying mechanisms are not fully understood (e.g. Brönnimann et al., 2019b). Before 1850, instrumental data are only available at limited locations and proxy records typically provide information at annual or lower resolution. Therefore, in many studies the different data types are combined to achieve a better performance. Multiple European reconstructions, for instance, combined instrumental measurements, historical evidence and proxy records (Luterbacher et al., 2004; Pauling et al., 2006; Küttel et al., 2010). These reconstructions with high spatial and temporal resolution can reveal regional differences and can identify distinct seasonal variability. However, such reconstructions are not available for the entire globe. Although not following the true evolution of past climate variability, model simulations provide information on forced and internal variability of various elements of the climate system at full spatial coverage. Therefore, they can contribute to better understand climate dynamics and related processes.

In addition to traditional reconstruction methods and climate models, data assimilation offers a new way to investigate past climate changes. In the paleoclimate data assimilation framework, past climate states are estimated from climate model simulations and observational information. The two sources of information are optimally combined, and the obtained reconstruction is regarded as the best estimate of the past climate. In the recent years, several studies explored different data assimilation methods to reconstruct past climate (e.g. Hakim et al., 2013, and references therein). For the climate of the last Millennium, the most used approaches rely on ensemble‐based Kalman filtering techniques, which were applied, for instance, in pseudo‐proxy experiments (e.g. Bhend et al., 2012), hydroclimate reconstructions (Steiger et al., 2018) or the generation of a monthly atmospheric paleo‐reanalysis (EKF400, Franke et al., 2017).

EKF400 is a global 3‐dimensional climate reconstruction, that spans the period between 1600 and 2005 (Franke et al., 2017). It was generated with the ensemble Kalman fitting technique (Franke et al., 2017), the offline variant of the ensemble square root filter (Whitaker & Hamill, 2002). It blends the ECHAM5.4 atmospheric model simulations called CCC400 (Bhend et al., 2012) with different types of observations (early instrumental measurements, documentary data, proxy records). EKF400 is the first paleo‐reanalysis that provides multivariate information about the state of the atmosphere during the last 400 years at monthly resolution. Such data are highly relevant for climate impact studies or to investigate the role of atmospheric circulation. It has been used, for instance, to study climate anomalies related to volcanic eruptions (Brönnimann et al., 2019b; Zambri, et al., 2019) and droughts (Burgdorf et al., 2019).

Given the novelty of EKF400, several further development possibilities were identified. This paper outlines the developments applied in the generation of the new version of EKF400 (EKF400v2). The observational network of previously assimilated data types is notably extended. In addition, in version 2 of EKF400, new data sources such as corals and precipitation information (amounts and number of wet days) were assimilated (Valler et al., 2020). Another line of improvements focuses on methodological developments. The new version of EKF400 takes advantages of improved covariance localization and the application of better estimated background‐error covariance matrix (Valler et al., 2019).

The aim of this paper is to describe the dataset, highlight the improvements over EKF400 version 1 (EKF400v1) and to inform the users about its limitations, so that it can be effectively used in future climate studies. This article is organized as follows. In Section 2, we describe the model simulations and the observational network used as well as the ensemble Kalman fitting (EKF) paleoclimate data assimilation method. In Section 3, the reconstruction experiment and the methodological improvements are introduced. The skill of EKF400v2 is analysed in the period between 1902 and 2002 in Section 4. In addition, two case studies of Central European droughts from 1726 to 1728, and the effect of La Niña in the 19th century is presented in Section 4. Finally, we draw our concluding remarks in Section 5.

## 2 DATA PRODUCTION METHODS

### 2.1 Data sources

#### 2.1.1 Model simulations

EKF400v2 uses the same model simulations (CCC400) as EKF400v1. The 30 ensemble members were generated with the ECHAM5.4 general circulation model (Roeckner et al., 2003). The model simulations cover the period between 1600 and 2005. Reconstructed solar irradiance, sea surface temperatures and land surface parameters, volcanic activity, concentrations of greenhouse gases and sulphate were used to run the model at T63 resolution with 31 vertical levels (described in more detail in Bhend et al., 2012). The forcings and boundary conditions used to produce the model simulations are summarized in Table 1. Some errors were detected in the representation of land surface classes, which mainly affected the temperature field over the land areas in the extratropical Northern Hemisphere, and unrealistic wind speeds were found at southern high latitudes (Franke et al., 2017; Rohrer et al., 2018). Hence, we recommend not to use the dataset south of 60°S. The 6‐hourly model output files were converted to monthly means. Several fields are updated by the assimilation: temperature at 2 m, precipitation, number of wet days, sea‐level pressure, horizontal wind components at 850, 500 and 200 hPa, vertical motion at 500 hPa, geopotential height at 1,000, 500, 300, 200 and 100 hPa, blockings and cyclone frequency. In version 2, we provide a dataset with 2° resolution globally, double the resolution of version 1.

**TABLE 1:** Summary of boundary conditions and forcings used for the CCC400 model simulations

Feature	References
sea surface temperature	Mann et al., (2009)
Niño‐3.4 index	Cook et al., (2008)
climatological sea‐ice	Rayner et al., (2003)
solar irradiance	Lean (2000)
land‐use	Pongratz et al., (2008)
volcanic forcing	Crowley et al., (2008)
greenhouse gases	as used in Yoshimori et al., (2010) and references therein
sulphate	Koch et al., (1999)

#### 2.1.2 Observational dataset

Various data types from proxy records to documentary data to instrumental measurements are involved in the assimilation.

In EKF400v1, only a small number of proxy records of tree‐ring width (TRW) and maximum latewood density (MXD) were used (Franke et al., 2017). In EKF400v2, additional tree‐ring proxy records were included from different databases such as the vast collection of tree‐ring width chronologies of Breitenmoser et al., (2014), the N‐TREND (Wilson et al., 2016) and the PAGES2k‐2017 (Emile‐Geay et al., 2017) data collections. In addition, selected tree‐ring chronologies from the collection by Neukom et al., (2014) were also added to the input data (Table 2).

**TABLE 2:** Input data used for EKF400v1 and EKF400v2

Data	Variable	Time	Data source	Reference
Anjarakandy*	T	1810–1823	inst	Dove (1847)
Archangelsk	T	1813–2001	inst	Brohan et al., (2006)
Barcelona*	T	1780–2004	inst	Barriendos et al., (1997)
Burnley*	R	1677–1704	inst	Townley, 1694, Townley, 1699), Derham (1705)
Cambridge* (US)	P	1742–1812	inst	Dupigny‐Giroux et al., (2007)
Carpathian basin	T	1601–1854	docu	Bartholy et al., (2004)
Central Belgium	T	1794–2001	inst	Demarée et al., (2002)
Central England	T	1659–2004	inst	Manley (1974), Parker et al., (1992)
Central Europe	T	1601–1854	docu, inst	Dobrovolný et al., (2010)
Central Europe (Germany)	T	1600–1759	docu	Glaser and Riemann (2009)
Charleston*	T, R	1738–1759	inst	Blodget (1857)
Edo (Tokyo)*	T	1643–1890	docu	Aono (2015)
Eastern Carpathians	T	1601–2004	proxy	Popa and Kern (2009)
Forfjorddalen	T	1601–2004	proxy	McCarroll et al., (2013)
Funchal (Madeira)*	R	1747–1753	inst	Alcoforado et al., (2012)
Funchal (Madeira)	T	1749–1802	inst	Alcoforado et al., (2012)
GHCN‐Daily v3.27*	NR	1781–2004	inst	Menne et al., (2012)
GHCN‐Monthly v2*	R	1697–2004	inst	Lawrimore et al., (2011)
GHCN‐Monthly v2	P	1755–2002	inst	Peterson and Vose (1997)
Gibraltar*	R	1812–2004	inst	Wheeler (2007)
Gibraltar	T	1821–2003	inst	Wheeler (2006)
Gordon Castle	P	1782–1827	inst	Buchan (1880)
gridded MXD	T	1601–2004	proxy	Briffa et al., (2004)
HISTALP	T	1760–2004	inst	Auer et al., (2007), Böhm et al., (2010)
HISTALP	P	1763–2004	inst	Auer et al., (2007)
HISTALP*	R	1800–2004	inst	Auer et al., (2007)
Ilulissat	T	1835–2004	inst	Vinther et al., (2006)
Ireland*	R	1711–2004	inst	Murphy et al., (2018)
ISTI v1.1.0*	T	1701–2004	inst	Rennie et al., (2014)
Jämtland	T	1601–2004	proxy	Gunnarson et al., (2011)
Kawanishi‐Yamagata*	T	1830–1980	docu	Ohba et al., (2013)
Kiev	T	1812–2001	inst	Brohan et al., (2006)
Kyoto*	T	1601–2004	docu	Aono and Kazui (2008)
Kyoto*	T	1601–1995	docu	Aono and Tani (2014)
Lapland	T	1601–1998	proxy	Helama et al., (2009)
London	P	1692–2004	inst	Cornes et al., (2012b)
MXD records	T	1601–2004	proxy	WSL (2019)
Nagasaki	P	1828–2000	inst	Können et al., (2003)
Nagasaki	T	1819–2000	inst	Können et al., (2003)
Naples*	T	1821–1846	inst	Dove (1847)
Nuuk	T	1784–2004	inst	Vinther et al., (2006)
Nyzhny Tagil	P	1840–1865	inst	KNMI (1871)
northern Fennoscandia*	T	1693–2004	docu	Loader et al., (2011)
N‐TREND*	T	1601–2004	proxy	Wilson et al., (2016)
PAGES2k‐2017*	T, R	1601–2004	proxy	Emile‐Geay et al., (2017)
Paris*	T	1658–2004	inst	Rousseau (2009, 2013)
Paris	P	1670–2004	inst	Cornes et al., (2012a)
Paris*	R	1688–2004	inst	Slonosky (2002)
Poland	T	1601–1696	docu	Przybylak et al., (2005)
Pressure collection	P	1749–2004	inst	Küttel et al., (2010)
Pyrenees	T	1601–2004	proxy	Büntgen et al., (2008)
Pyrenees	T	1601–2004	proxy	Linán et al., (2012)
Qaqortoq	T	1807–2004	inst	Vinther et al., (2006)
Rio de Janeiro*	T	1832–1843	inst	Dove (1847)
Rio de Janeiro*	R	1781–1788	inst	Farrona et al., (2012)
Rio de Janeiro	T	1781–1788	inst	Farrona et al., (2012)
Salem (Massachusetts)	P	1786–1820	inst	Van Der Schrier and Jones (2008)
San Fernando (Cadiz)*	R	1805–1944	inst	Barriendos et al., (2002)
San Fernando (Cadiz)	T	1787–1996	inst	Barriendos et al., (2002)
Savanna‐la‐Mar (Jamaica)*	R	1760–1786	inst	Chenoweth (2003)
Savanna‐la‐Mar (Jamaica)	T	1764–1776	inst	Chenoweth (2003)
Stockholm	P	1756–2004	inst	Moberg et al., (2002)
St. Lawrence Valley*	T	1742–2004	inst	Slonosky (2014)
St. Petersburg	T	1743–2004	inst	Jones and Lister (2002)
Switzerland	T	1601–1816	docu	Pfister (1999)
Switzerland	T	1601–2004	proxy	Büntgen et al., (2006)
Tatra	T	1601–2004	proxy	Büntgen et al., (2013)
Tokyo (Hachioji) *	T	1721–1941	docu	Mikami (1996)
Tokyo	T, P	1839–2000	inst	Zaiki et al., (2006)
Tomsk*	T	1830–1838	inst	Dove (1847)
Torneträsk	T	1601–2004	proxy	Grudd (2008)
Tree and coral records*	T, R	1601–2004	proxy	Neukom et al., (2014)
TRW collection*	T, R	1601–2004	proxy	Breitenmoser et al., (2014)
Tyrol	T	1601–2003	proxy	Esper et al., (2007b)
Uppsala	T	1722–2000	inst	Bergström and Moberg (2002)
Yokohama	T	1860–2000	inst	Zaiki et al., (2006)
Zafra*	T	1750–1839	docu	Fernández‐Fernández et al., (2014)

Note

In the Data column, the location of single time series as well as data compilations used in the input file are presented. In the Variable column, it is indicated which variable(s) the observation represent, where NR denotes number of wet days, P is pressure, R is precipitation, and T is temperature. In the Time column, the period relevant for the assimilation is shown. The data can come from documentary (docu), instrumental (inst) and proxy sources. A reference for each time series or data collection is given in the last column.

* Denotes newly added time series /databases in EKF400v2.

**FIGURE 1:**
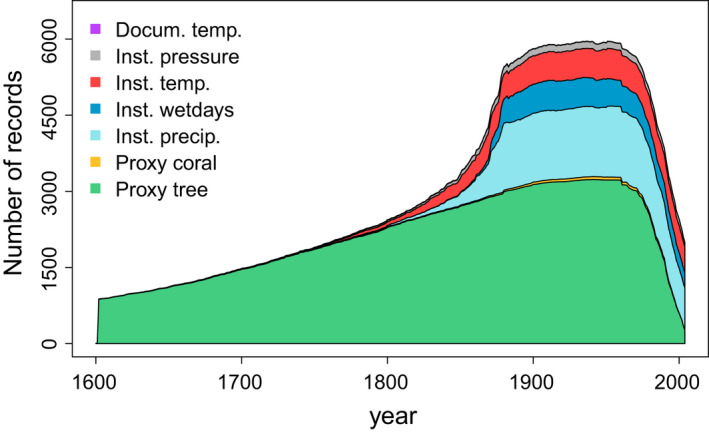
Summary of different input data types and variables per year. The documentary temperature (Docum. temp.) series (purple line) is hardly visible because only 12 series are included in the input file (see Table 2)

In version 2 of EKF400, assimilated proxy types were further extended with the assimilation of corals. Corals have annual to sub‐annual resolution, which is ideal for our monthly resolved reconstruction and they cover low‐latitude regions, where trees cannot serve as temperature proxies. Sub‐annually resolved coral chronologies from the PAGES2k‐2017 database are used in EKF400v2. The proxy input data file has 3,513 entries in total, which means more than 20 times more proxy data compared to EKF400v1 (Figure 1).

Temperature information from documentary data was also assimilated in EKF400v1. The five documentary data from Central Europe provide monthly information about the climate including the winter (or non‐growing) season, an advantage compared to proxy records. In the input file of EKF400v2, two further documentary data series from Europe (Fernandez‐Fernandez et al., 2014; Loader et al., 2011) and five new data series from Japan (Aono, 2015; Aono & Kazui, 2008; Aono & Tani, 2014; Mikami, 1996; Ohba et al., 2013) are included, leading to a total number of 12 documentary data series.

In the EKF400v1, most of the temperature time series were obtained from the Global Historical Climatology Network‐Monthly version 3 dataset (GHCN‐Monthly v3, Lawrimore et al., 2011), with the earliest series starting in 1801. Because the main focus of the paleo‐reanalysis is the pre‐20th century period, only series starting before 1,880 were kept. In EKF400v2, GHCN‐Monthly v3 is replaced with the merged data collection of the international surface temperature initiative (ISTI, Rennie et al., 2014). Like in the case of GHCN‐Monthly v3 data, time series that start before 1880 were selected, leaving 619 records. The earliest series in the ISTI database starts in 1701. The additional time series, which were included in EKF400v1 from the Historical Instrumental Climatological Surface Time series of the Greater Alpine Region database (HISTALP, Auer et al., 2007; Bohm et al., 2010) and separately collected series from various sources (Franke et al., 2017), were also included in EKF400v2, together with eight further stations from Brazil (Dove, 1847), Canada (Slonosky, 2014), France (Rousseau, 2009; Rousseau, 2013), India (Dove, 1847), Italy (Dove, 1847), Russia (Dove, 1847), Spain (Barriendos et al., 1997) and the United States (Blodget, 1857) (Table 2). Altogether, the temperature input file contains 701 time series.

The GHCN‐Monthly dataset version 2 contains precipitation data of several thousands stations (Lawrimore et al., 2011), from which the stations where measurements started before 1880 were included in the assimilation (1,437 stations). From the HISTALP data collection, 69 precipitation time series were used. In addition, 9 time series from Brazil (Farrona et al., 2012), England (Derham, 1705; Townley, 1699; Townley, 1694), France (Slonosky, 2002), Gibraltar (Wheeler, 2007), Ireland (Murphy et al., 2018), Jamaica, (Chenoweth, 2003), Madeira (Alcoforado et al., 2012), Spain (Barriendos, et al., 2002) and the United States (Blodget, 1857) were added to the assimilated data (Table 2). In total, the precipitation input file contains 1515 stations data. In addition to monthly precipitation amounts, the number of wet days was also included in the assimilation. Wet days records were calculated from the GHCN‐Daily dataset version 3.27 (Menne et al., 2012). A daily precipitation amount ≥1 mm was considered a wet day. A total of 652 wet days series are included, the earliest starting in 1781.

In the input file of EKF400v1, pressure time series were obtained from GHCN‐Monthly v2 (Peterson & Vose, 1997), from HISTALP, from the collection made by Kuttel et al., (2010) and additional individual records were included (Franke et al., 2017). In EKF400v2, the pressure time series were completed with one additional station from the United States (Dupigny‐Giroux et al., 2007). The pressure input file contains 141 time series.

**FIGURE 2:**
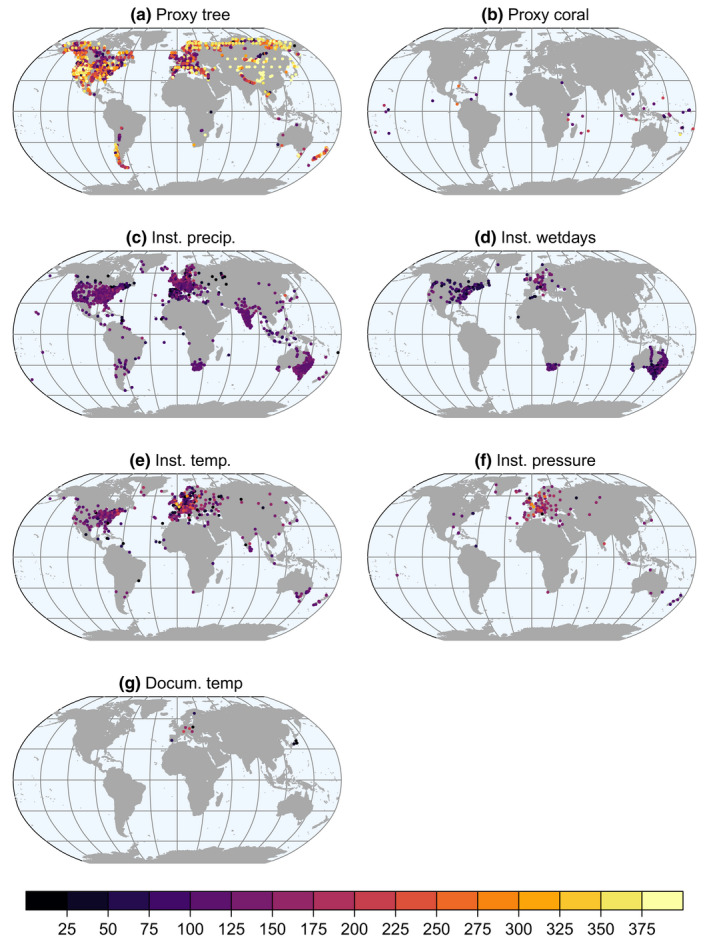
Spatial distribution of the input data types. The colours indicate the length of each series in years

An overview of the number of records per year and the spatial distribution of observation types are shown in Figures 1 and 2. The vast majority of the data are tree‐ring width and density records throughout the reconstruction period. In Europe, a few long instrumental measurement series are available from the second half of the 17th century. In the 20th century, there are around 6,000 data entries. However, the input data file differs from the assimilated data because the data have to pass several preprocessing steps before they get assimilated (for more details, see Section 3).

### 2.2 Ensemble Kalman fitting

The ensemble Kalman fitting method (EKF) uses a transient offline data assimilation approach. In this offline approach, an ensemble of precomputed transient model simulations, which follow reconstructed forcings and boundary conditions, are combined with the observations. In our case, the CCC400 model simulations are blended with the above described observational data. Applying an offline scheme is a common practice for paleoclimate reconstruction because as argued in Bhend et al., (2012) predictability comes from the external forcings and boundary condition prescribed to the model simulations. Moreover, the offline approach allows simple testing of changes applied to the method since the simulations do not have to be recalculated.

The model and observational information are combined in the EKF following the procedure proposed by Whitaker and Hamill (2002); the ensemble mean ($\bar{x}$) and the ensemble members, transformed to deviations from the mean ($x_{i}^{\prime }=x_{i}‐\bar{x}$), are updated separately:

(1) $${\bar{x}}^{a}={\bar{x}}^{b}+K\left(y‐H{\bar{x}}^{b}\right)$$

(2) $$x^{\prime a}=x^{\prime b}+\tilde{K}\left(Hx^{\prime b}\right)$$

where *x^a^* denotes the analysis and *x^b^* is the background state vector, obtained from the model simulation. *y* represents the observations. **H** is the forward operator, that connects the model space with the observation space. In our setup, **H** depends on the assimilated variable but it is always linear. **K** and $\tilde{K}$ are the Kalman gain matrix and the reduced Kalman gain matrix:

(3) $$K=P^{b}H^{T}{\left({\mathrm{HP}}^{b}H^{T}+R\right)}^{‐1}$$

(4) $$\tilde{K}=P^{b}H^{T}{\left({\left(\sqrt{{\mathrm{HP}}^{b}H^{T}+R}\right)}^{‐1}\right)}^{T}\times {\left(\sqrt{{\mathrm{HP}}^{b}H^{T}+R}+\sqrt{R}\right)}^{‐1}$$

The Kalman gain matrix based on the error estimates of the model (**P^b^**) and of the observation (**R**) determines how to incorporate the observation into the model. **P^b^** is the background‐error covariance matrix, which in the case of ensemble‐based methods is calculated from the perturbations of the ensemble members. **R** is the observation‐error covariance matrix, and it is assumed that its elements are uncorrelated.

The EKF is implemented to process observations serially, which simplifies the assimilation process (Whitaker & Hamill, 2002). Due to the small ensemble size (30 members), the background‐error covariance matrix is localized in order to prevent long‐range spurious correlations. We apply a localization method, in which **P^b^** is multiplied element‐wise with a distant‐dependent correlation function:

(5) $$G=\exp\left(‐\frac{z^{2}}{2L^{2}}\right),$$

where *z* is the distance in km between two grid boxes and *L* is the length‐scale parameter. The length‐scale parameters used in the localization were calculated from the CCC400 model simulations for each variable (see Table 1 in Franke et al., 2017). The cross‐covariances are localized by applying the smaller *L* values of the two variables in Equation (5).

In the newly generated climate reconstruction, **P^b^** is replaced with **P^blend^** to better represent the covariances (for more details see Section 3).

## 3 OVERVIEW OF THE ASSIMILATION PROCESS

In the production of the new reconstruction (EKF400v2), the basic features are consistent with the experimental design of EKF400v1. The assimilation is conducted on anomaly level. Hence, both the model simulations and the observations are transformed to anomalies calculated from a 71‐year running climatology of the model and the observations. The climatology used for calculating the anomaly is shorter at the ends of the reconstructed period. We chose this procedure because the multi‐decadal to centennial‐scale variability may not be preserved in many proxy records in the collections used here (e.g. Franke et al., 2013; Tardif et al., 2019). This way low frequency variability is consistent with the model response to external forcings. The assimilation window is 6 months long, lasting from October to March and from April to September. The assimilation window was chosen in accordance with the growing season of trees, to assist the assimilation of time‐averaged proxy records. Therefore, *x^b^* contains 6 months data of variables of interest.

The observational network is substantially extended by including new datasets and newly digitized data, as well as adding new data types such as precipitation time series and coral proxy records to the assimilated data. The observations are quality checked before they are assimilated as in EKF400v1. Proxy records and instrumental measurements are excluded if they are more than five standard deviations away from their mean in the current time step.

Although proxy records are still assimilated by creating a forward operator using a multiple regression technique in EKF400v2, several modifications have been made. Tree‐ring series are calibrated between the 1901‐1971 period. In the new version, temperature‐sensitive proxy series are calibrated with the Berkeley Earth surface temperature data (Rohde et al., 2013) instead of the Climatic Research Unit's gridded time‐series version 3.10 dataset (CRU TS3.10, Harris et al., 2014) because of ocean coverage and higher spatial resolution. The MXD proxies are removed from the PAGES2k‐2017 dataset because they are already included in the other MXD collections. The remaining tree‐ring proxy records in the PAGES2k‐2017 and TRW collection of Breitenmoser et al., (2014) can be both temperature‐ and precipitation‐sensitive (Franke et al., 2020a). Therefore, to relate the proxy signal with instrumental measurements, for precipitation calibration the CRU TS4.03 (Harris & Jones, 2019) and for temperature calibration the Berkeley Earth surface temperature data are used. The regression model is built from 1 to 6 subsequent months of temperature variables in the case of temperature‐sensitive proxies and from both temperature and precipitation variables in the case of temperature‐ and precipitation‐sensitive proxies. Hence, a maximum of 12 explanatory variables are included in the multiple linear regression model, which would theoretically be the case when a tree would respond to temperature and precipitation during the entire growing season. (In our multiple linear regression models, we found no considerable multicollinearity among the explanatory variables, which could lead to less stable models.) The optimal way of assimilating various tree‐ring databases was investigated in a previous study (Franke et al., 2020a). Based on the results of the experiments, the most skilful reconstruction was obtained when all databases were combined and the assimilation of proxy records was preceded with a careful quality check, discarding proxy records with insignificant climatic information. Therefore, following their findings proxy records are assimilated only if they contain a clear climatic signal. To assess whether a proxy record has a significant climatic signal, an F‐Test (Neter et al., 1996) is conducted and the null‐hypothesis (all of the regression coefficients are equal to zero) must be rejected with *p* < 0.05; otherwise, the proxy record is excluded. Furthermore, tree‐ring proxies are screened to remove possible duplicates by checking for multiple records in a 0.1° × 0.1° grid box, and if more than one proxy belongs to the same grid box, only the one with the smallest residual is kept.

In the PAGES2k‐2017 database, coral oxygen‐isotope and Sr/Ca signals are available at sub‐annual resolution. Because we only have δ^18^O information for all corals and only rarely additional Sr/Ca measurements, we use a simple approach and treat corals as only being temperature‐sensitive. The regression coefficients for coral records are calculated using Berkeley Earth, which also provides information from oceanic regions by including the Met Office Hadley Centre sea surface temperature (HadSST) dataset (Kennedy et al., 2011). Since corals grow all year round, seasonal averages are created from the sub‐annual coral data of the PAGES2k‐2017 database (Emile‐Geay et al., 2017) for the October‐March and April‐September time periods. The aggregated seasonal values are calibrated with the HadSST data between October 1901 and September 1971. Therefore, a maximum of 6 regression coefficients are obtained from the multiple linear regression, if corals respond to sea surface temperature in the entire season. Coral records are treated as surface temperature observations under the assumption that the surface water in which the corals grow is closely related to air temperature above.

Instrumental and documentary data are assimilated as described in Franke et al., (2017). The **H** operator extracts the closest grid cell to the observation location from the model simulations which is then used in the assimilation process. Instead of using a horizontal interpolation, we averaged the data per grid cell if several same type of observations belonged within one grid box (smoothing *y*). We estimated instrumental temperature and sea‐level pressure observations errors based on the methods of Desroziers et al., (2005) and Wartenburger et al., (2013). We found similar errors estimates as were used by Franke et al., (2017) in a previous study. The prescribed observational error of instrumental temperature and sea‐level pressure measurements are √0.9 K and √10 hPa, respectively. The error in instrumental precipitation measurement is estimated as 30% of the measured data or at least 10 mm, and the estimated error in the number of wet days is 2 days (Valler et al., 2020). The assigned error of documentary data is 0.5 standard deviations. The error of proxy records is estimated with the residuals of the multiple regression fit. Possible duplicates in the instrumental data were not removed; however, the same type of instrumental observations are averaged if more than one are located in the same grid box. In the case if both documentary and instrumental data are available from the same location, only the instrumental measurement is assimilated. However, proxy records are used in all cases, because they get substantially less weight due to their larger errors.

The background‐error covariance matrix in EKF400v1 is calculated from the 30 ensemble members and localized with the previously defined *L* values (Franke et al., 2017). The background‐error covariance matrix plays a key role in the assimilation; therefore, it is essential to estimate it correctly. Valler et al. (2019) tested different methods for improving the estimation of **P^b^**. Based on those results, in EKF400v2 the background‐error covariance matrix is not only estimated from the sample covariance but combined with a climatological covariance matrix (**P^clim^**). First, we create a climatological state vector (*x* ^clim^) by randomly selecting 100 years from the 405‐year long 30‐member ensemble runs; that is, the climatological state vector consists of 100 random atmospheric states. **P^clim^** is calculated from this climatological state vector (Valler et al., 2019). In the new reconstruction, *x* ^clim^ is recreated every year from 100 randomly selected members. The two covariance matrices are combined as:

(6) $$P^{\text{blend}}=\beta_{1}P^{b}+\beta_{2}P^{\text{clim}},$$

where β_1_ and β_2_ are the weights given to the covariance matrices and **P^blend^** denotes the blended covariance matrix. Because **P^clim^** is calculated from a larger ensemble, higher *L* values are applied for its localization. In the reconstruction experiment, we double the *L* values used for localizing **P^clim^** and weight the two covariance matrices equally (β_1_ and β_2_ are 0.5). This setup has been shown to improve the skill of the reconstruction (Valler et al., 2019). Hence, EKF400v2 is generated by replacing **P^b^** with **P^blend^** in Equations (3) and (4). Additionally, as found in previous sensitivity experiments, the reconstruction had the highest skill when both *x^b^* and *x* ^clim^ were updated with the observational information (Valler et al., 2019); thus, the same procedure was applied here as well. Since the order of observations may affect the reconstruction (e.g. Greybush et al., 2011), we assimilate them in the following order: (1) instrumental measurements (temperature, pressure and finally precipitation); (2) documentary data; (3) proxy records.

In addition to the above‐mentioned technical modification, the covariance matrix is also localized in time. It is a special feature of the EKF method, which is necessary due to combining several months into one assimilation window (Valler et al., 2019). Time localization is applied to monthly instrumental and documentary data by setting all cross‐months covariances to zero. Therefore, these observations only influence the model fields of the current month.

## 4 EVALUATING THE SKILL OF EKF400v2

### 4.1 Skill analysis in the 20th century

The EKF400v2 reconstruction is compared first to the CCC400 model simulations, and later, a comparison between version 1 and version 2 of EKF400 is also shown. A leave‐one‐out validation was already carried out for version 1, and this is one reason why we show the improvements over version 1. We investigate the skill of the analysis ensemble mean (${\bar{x}}^{a}$) by using the Berkeley Earth surface temperature data (Rohde et al., 2013), the CRU TS4.03 (Harris & Jones, 2019) and the Hadley Centre's monthly historical mean sea‐level pressure (HadSLP2, Allan & Ansell, 2006) datasets as references for temperature, precipitation and sea‐level pressure evaluation, respectively. The EKF400v2 is not fully independent from these data products mainly because of the assimilated instrumental measurements which start before 1880 and may have been included in the gridded instrumental datasets as well. Regression coefficients of the proxy forward models are estimated from the Berkeley Earth and the CRU TS4.03 datasets, which are used to map model data to proxy records; the errors of proxy records are estimated with the regression residuals. As a consequence, the absolute skill of the reconstruction is likely overestimated. Hence, we also compare the skill to the previous version to better capture the improvements and use newly digitized temperature series for independent evaluation.

**FIGURE 3:**
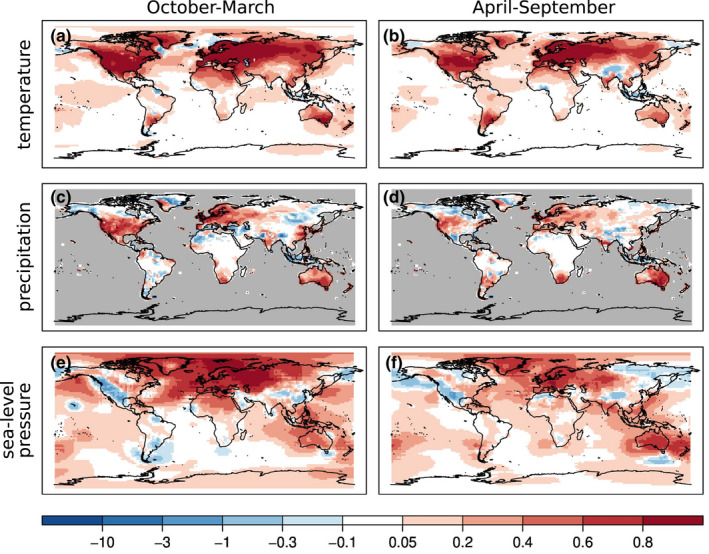
Spatial distribution of RMSESS values of temperature (a, b), precipitation (c, d) and sea‐level pressure (e, f) in the two seasons. The RMSESS is calculated using the ensemble mean of the analysis and the model simulation as well as gridded instrumental data as reference. The grey shaded areas indicate the region where no reference data are available

Although our reconstruction has monthly resolution, the skill metrics shown in this Section depict seasonal averages calculated over the October‐March (winter) and April‐September (summer) time periods between 1902 and 2002. Figure 3 shows the correlation coefficient differences between the correlations calculated from the analysis and the reference data and the background state vector and the reference data. Correlation differences are shown because the forced model simulations already correlate with instrumental observations. Correlation differences are almost always positive for all the three variables in both half‐years. The largest improvement in the temperature field is over the Northern Hemisphere, where most of the observations are located. The improvement over Europe, especially in summer (Figure 3b) and over India in winter (Figure 3a) is smaller, because the model already performs relatively well in these regions (Figure S1). Correlation increases over the central Pacific Ocean, presumably due to the assimilation of coral records. Notable increases in the correlation coefficients of precipitation are seen in Figure 3c and d. The increase is more concentrated around the instrumental precipitation measurements. The larger increase is also due to the relatively worse performance of the model in simulating precipitation accurately. The sea‐level pressure reconstruction shows improvement mainly over the North Atlantic region and Australia, where plenty of pressure measurements are available.

**FIGURE 4:**
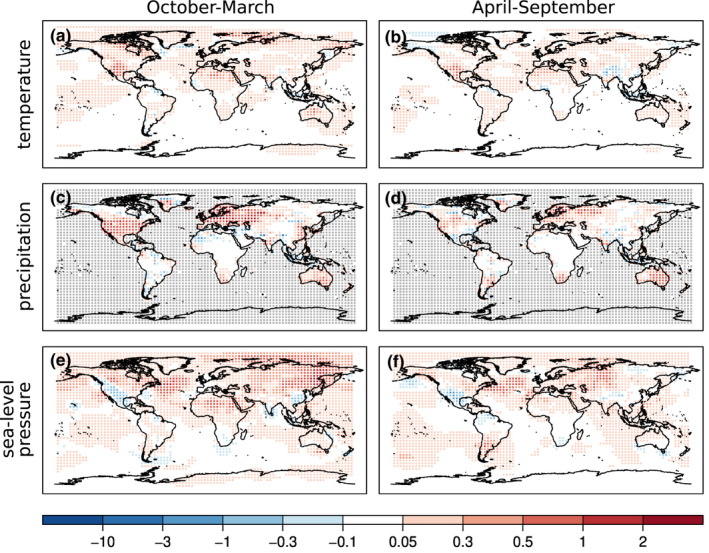
Spatial distribution of RMSESS values differences between EKF400v2 and EKF400v1 (EKF400v2 minus EKF400v1). The differences are calculated on the resolution of EKF400v1 for temperature (a, b), precipitation (c, d) and sea‐level pressure (e, f) in the two seasons. Positive values imply that v2 has higher skill than v1. The grey shaded areas indicate the region where no reference data are available. Note that the colour scale is nonlinear and asymmetric

Figure 4 shows the reconstruction skill in terms of the root mean square skill score (RMSESS, Wilks, 2011). While correlation expresses the covariability between two time series, RMSESS also rewards correct estimation of the amplitude. RMSESS is calculated as:

(7) $$\text{RMSESS}=1‐\frac{\sqrt{\frac{1}{n}\sum_{i=1}^{n}{\left(x_{i}^{u}‐x_{i}^{\text{ref}}\right)}^{2}}}{\sqrt{\frac{1}{n}\sum_{i=1}^{n}{\left(x_{i}^{f}‐x_{i}^{\text{ref}}\right)}^{2}}},$$

where in our case, *x^u^* is the ensemble mean of the reconstruction (${\bar{x}}^{b}$), *x^f^* is the ensemble mean of the model simulation (${\bar{x}}^{b}$), *x* ^ref^ is the reference dataset, and *i* refers to the time step. The RMSESS is calculated from the 71‐year anomalies. Positive RMSESS values indicates improvement over the CCC400 model simulation ensemble mean, while RMSESS = 1 means that the reconstruction is equal to the reference data (a perfect reconstruction). In previous publications, the RMSESS was termed reduction of error (RE; e.g. Franke et al., 2017).

The RMSESS values of temperature are mainly positive (Figure 4a, b). As expected, the largest improvement is over the land areas in the Northern Hemisphere, but the reconstruction also shows some skill over oceanic areas. The skill of the reconstruction is very similar in both seasons in the 20th century when instrumental data are available.

The RMSESS values of precipitation depict more spatial variability (Figure 4c, d). Over Europe, India, Australia and South Africa, the skill is positive both in winter and in summer. The precipitation reconstruction performs better over North and Central America in the October‐March half‐year. Similarly, the reconstruction is more skilful in the April‐September period over the southern part of South America. In the sea‐level pressure reconstruction, the largest improvement occurred over the North Atlantic sector and Eurasia, with more notable improvement in the October‐March period. In the Southern Hemisphere, the improvement is also more pronounced during winter time from April to September, but the degree of improvement is smaller. However, negative RMSESS values characterize, for example, the North American Cordillera area in both half‐years.

**FIGURE 5:**
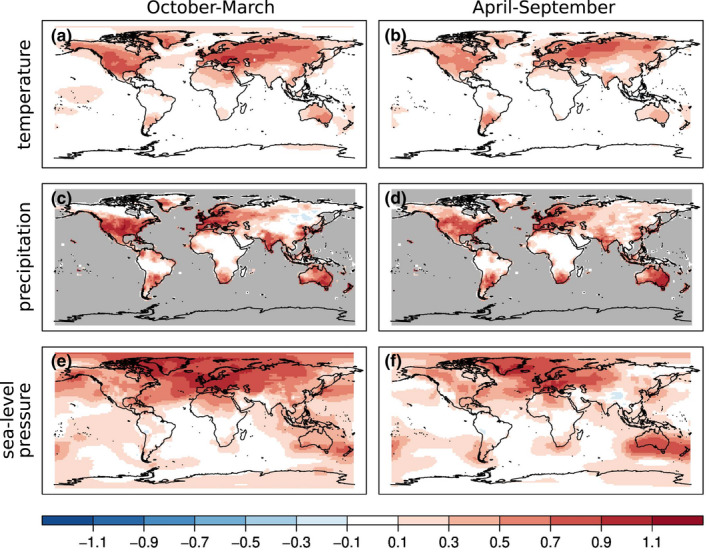
Spatial distribution of correlation coefficients differences between the analysis mean of EKF400v2 and the ensemble mean of the forced simulations of temperature (a, b), precipitation (c, d) and sea‐level pressure (e, f) in the two seasons. The grey shaded areas indicate the region where no reference data are available

To investigate the improvement of version 2 in comparison to version 1, the RMSESS of both versions are calculated and their differences (v2 minus v1) are shown in Figure 5. Since EKF400v1 was generated by using every second grid box in both the latitude and longitude, the comparison was made on the resolution of EKF400v1.

The reconstructed temperature field already had very high RMSESS values over the Northern Hemisphere in EKF400v1 (see Figure 8 in Franke et al., 2017), but even in this region further improvements can be seen (Figure 5a, b). Large differences in RMSESS values of the precipitation occur between the two versions, especially over the extratropical Northern Hemisphere in the October‐March period (Figure 5c). However, the skill is not always positive, for example over North Africa in winter or over North India and the Tibetan plateau in summer. The reconstructed sea‐level pressure field also mainly exhibits improvements over EKF400v1. Large areas both over land and ocean are characterized by positive RMSESS values.

**FIGURE 6:**
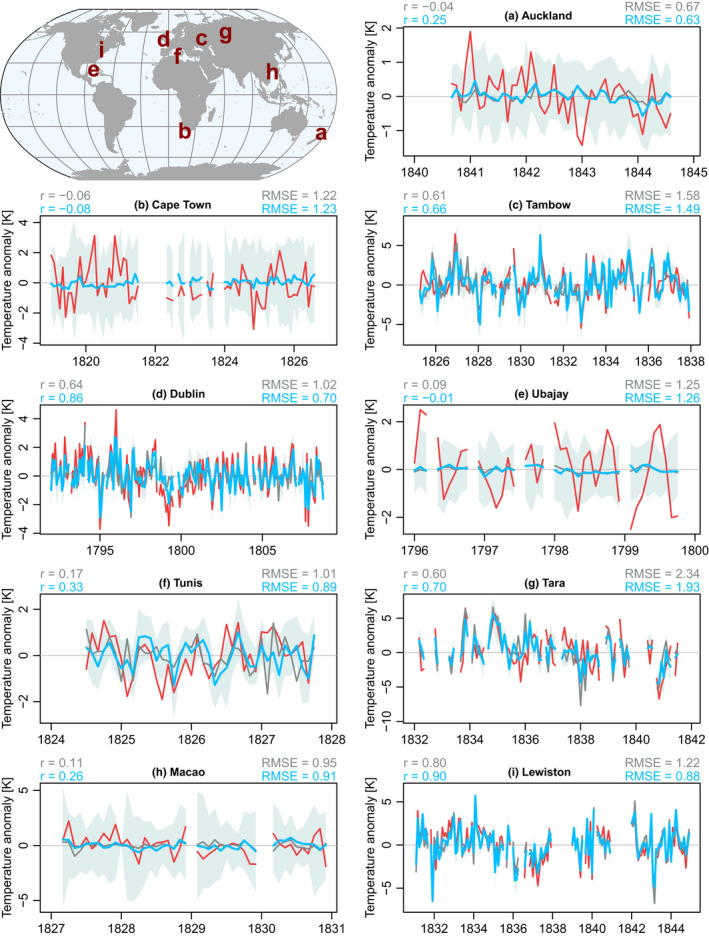
Monthly temperature anomalies of the independent observations (red), the analysis ensemble mean of EKF400v1 (grey) and EKF400v2 (blue). With light blue, the ensemble range of EKF400v2 is shown. The correlations (*r*) and the root mean square error (RMSE) between the reconstructions and the independent measurements are given on the figures. On the map, the location of observations is presented

Prior to the 20th century, the skill of reconstructed temperature field was evaluated with newly digitized data mainly from the 19th century (Dove, 1847; Weselowskij, 1857), which will be assimilated in the next version. Given that most of the new data come from coastal stations, we compared the independent time series with the analysis ensemble mean of the closest grid cell that we considered sufficiently representative of land surface temperature and that is present both in v1 and v2; for this reason, the selected grid cell can be up to 200 kilometres away from the location of the observations. The data at high northern latitudes show strong correlation with EKF400v1 but they further increased with EKF400v2 (Figure 6c,d,g,i). In the low latitudes, some improvement can still be seen in EKF400v2 compared to EKF400v1, although the uncertainty of the reconstruction there remains rather large (Figure 6e,f,h). In the cases of the two examples from the Southern Hemisphere, no nearby observations were assimilated in EKF400v1 over the analysed periods and no correlation is found with the independent temperature measurements (Figure 6a,b). In EKF400v2, tree‐ring proxies from the Southern Hemisphere were added to the assimilated observations which can affect the growing season climate fields; indeed, we can see some increase in the correlation between the Auckland temperature series and EKF400v2 (Figure 6a). However, no improvements appear for Cape Town (Figure 6b). Tree growth in South Africa is mostly limited by moisture and not temperature which could explain why the reconstructed temperature of EKF400v2 has not been improved by the assimilation.

### 4.2 Case studies

In order to further evaluate the skill of EKF400v2 in the pre‐20th century time, two case studies are investigated. We chose one summer‐ and one wintertime case study. In the summertime case study, we analyse a drought period over central Europe in the early 18th century and evaluate the reconstruction skill with independent early instrumental measurements that were not assimilated. In the wintertime case study, we look at known large‐scale teleconnection patterns by comparing the 19th‐century patterns with their known 20th‐century counterpart under the assumption of stationarity.

#### 4.2.1 Summertime example: Central European drought of 1726‐1728

According to documentary evidence, the late 1720s coincide with one of the driest periods in recorded history in Central Europe. Brázdil et al., (2013), for instance, reconstructed drought periods in the Czech Lands between 1501‐1804 and found the 1720s to be the decade with the most intense drought over the whole period. In 1726, in particular, six consecutive ‘dry’ months (April to September) were described in historical documents, although the impact on the population was more evident in 1727 (only June not classified as dry) and 1728 (whole summer ‘very dry’) with famine and even a rare locust plague (Brazdil et al., 2019).

**FIGURE 7:**
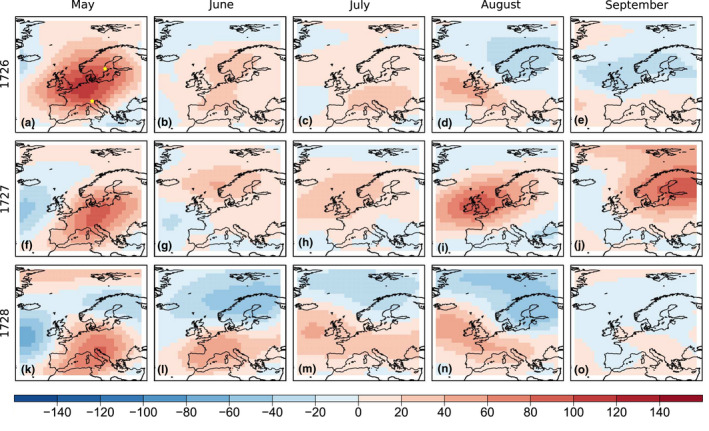
Monthly anomalies of 500 hPa geopotential height from May to September over Europe for the dry summer years between 1726 and 1728 in EKF400v2. The yellow stars indicate the location of Padua and Uppsala

This is also a decade in which early instrumental observations were carried out in several European cities, although only few of them have been recovered (Brönnimann et al., 2019a). Here we use the pressure data of Uppsala (Bergstrom & Moberg, 2002) and Padua (Camuffo et al., 2006), which have not been assimilated in either version of EKF400, to validate our reconstruction of the May to September periods in 1726, 1727 and 1728. The locations of the two stations are shown in Figure 7.

**FIGURE 8:**
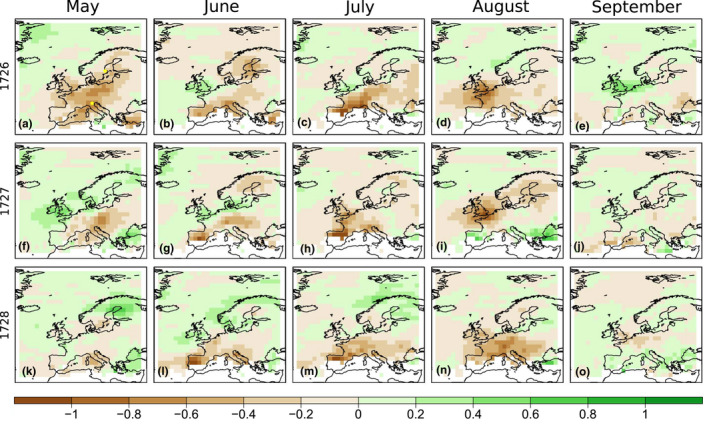
Monthly relative anomalies of precipitation from May to September over Europe for the dry summer years between 1726 and 1728 in EKF400v2. The areas left blank in the Mediterranean are regions where monthly precipitation amount is less than 10 mm in the climatology

Figures 7 and 8 show the reconstructed 500 hPa geopotential height and relative anomalies of precipitation fields over Europe in EKF400v2 for each month (equivalent figures for EKF400v1 are provided in the Supplement (Figures S2, S3)). As expected, most panels show negative precipitation anomalies in Central Europe, often reaching −60%. The anomalies of the geopotential height indicate a similar pattern in May in all three years, with an anomalous ridge over the continent. Weaker positive anomalies are reconstructed for most of Europe in nearly all months with the exception of September 1726 and 1728. These results clearly suggest drought conditions.

**FIGURE 9:**
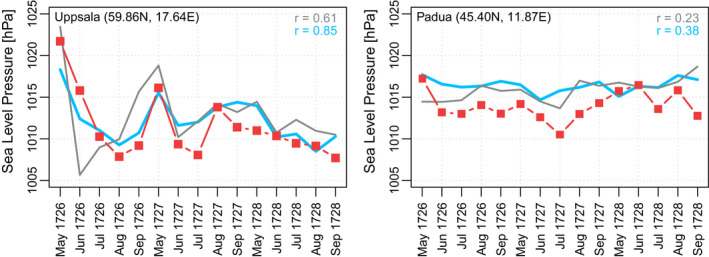
Monthly time series comparison among EKF400v1 (grey), EKF400v2 (blue) and instrumental pressure measurements (red) between 1726 and 1728. The correlations between the reconstructions and the independent measurements are given on the figures

The instrumental pressure records are compared with EKF400v1 and EKF400v2 in Figure 9. The correlation between observations and reconstruction is markedly higher for both stations in the new version. In both versions, only one pressure record (London, UK) is assimilated in that period. The skill increase can be due to the assimilation of precipitation as well as the additional tree‐ring width records and methodological upgrades.

#### 4.2.2 Wintertime example: La Niña impacts in the 19th century

**FIGURE 10:**
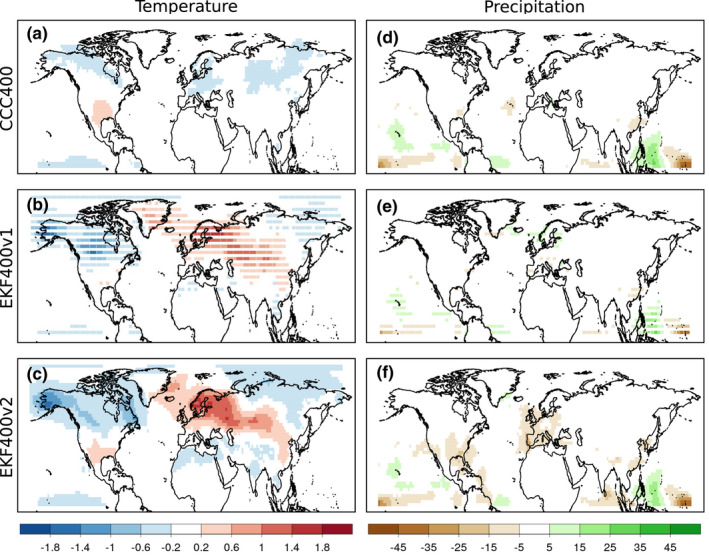
Composite figure of temperature anomaly [K] and precipitation anomaly [mm], calculated from the La Niña years in the 19th century (1801, 1820, 1822, 1835, 1842, 1847, 1863, 1872, 1887, 1890, 1893; based on Brönnimann et al., (2007)). The composite of ensemble mean of the model simulation (a, d), the EKF400v1 (b, e) and the EKF400v2 (c, f) are shown

The El‐Niño Southern Oscillation (ENSO) is known to have significant impacts on extratropical circulation (e.g. Livezey et al., 1997). In the mid‐latitudes of the Northern Hemisphere, many of the impacts are largest in late winter (January to March, or JFM) (e.g. Brönnimann et al., 2007; Schmidt et al., 2001). In order to better capture the ENSO signal (in this case, La Niña), a composite of all La Niña years in the 19th century (1801, 1820, 1822, 1835, 1842, 1847, 1863, 1872, 1887, 1890, 1893 (based on Brönnimann et al., 2007)) was calculated. In EKF400v1, the reconstruction skill for winter months in the 19th century was greatly limited by the number of observational and documentary records, particularly outside of Europe. As shown in Figure 10, the ability of the reconstruction to reproduce the La Niña signal in North America was practically equivalent to the ability of the model simulation. In version 2, numerous precipitation and temperature series are added in North America thanks to the assimilation of the GHCN‐Monthly v2 precipitation and ISTI datasets (see Table 2). This brings a clear improvement in the reconstruction of the ENSO signal in the south‐eastern United States, where La Niña conditions are related to strong negative precipitation anomalies (Schmidt et al., 2001). Assuming the stationarity of ENSO impacts on centennial scale (the La Niña composites of temperature and precipitation in the 20th century calculated from EKF400v2 are shown in Figure S4), positive temperature anomalies in the northern Gulf of Mexico (Livezey et al., 1997) are also more realistically reconstructed. ENSO also has an impact on the climate of Europe, which in the case of a La Niña event resembles to the positive North Atlantic Oscillation signal in Europe (Brönnimann et al., 2007). This signal was already well captured in version 1, although negative precipitation anomalies over Southern and Western Europe are more prominent in version 2.

## 5 SUMMARY

EKF400v2 was generated on the full spatial resolution of the model simulations, therefore it provides higher spatial resolution than EKF400v1. Over the 1902‐2002 period, generally high skill characterizes the EKF400v2 reconstruction but skill varies with the reconstructed variables, seasons and regions. An overall improvement in the skill of the three key variables (temperature, precipitation, sea‐level pressure) between the two versions has been found. In the early period, when primarily proxy data are available, most information is added to the summer growing season. To assess the skill prior to the twentieth century, we have looked at two case studies using independent data for evaluation. In the case of the summertime drought analysis, by investigating other unobserved variable fields of the state vector such as the 500 hPa geopotential height we can gain understanding of the climate dynamics leading to the extreme dry period. The skill of the reconstruction highly depends on the number of available observations. Further back in time, especially in the winter season, when only a few data series from Europe and Japan are assimilated, EKF400v2 is mainly driven by the model simulations.

## 6 DATASET LOCATION AND USAGE

The EKF400v2 monthly climate paleo‐reanalysis is archived at the World Data Center for Climate (WDCC) at DKRZ, and each ensemble member and the ensemble mean are stored in separate NetCDF files (Franke et al., 2020b). The files contain the absolute values of the following variables: air temperature at 2 m, total precipitation, sea‐level pressure, geopotential height at 500 and 100 hPa, eastward and northward wind components at 850 and 200 hPa and vertical velocity at 500 hPa. Data are accessible to everybody after registration.

The preliminary results with EKF400v2 highlight areas of improvement over EKF400v1 and can be used to investigate the dynamics behind past climate changes over the last 400 years. However, users must take into account the limitations of the dataset, the skill of the reconstruction changes with the regions and with the course of time. In places and times when assimilated data are abundant, the paleo‐reanalysis can be used with more confidence than, for instance, in the beginning of the reconstruction period when only proxy records are available. Since the assimilation was conducted assimilating anomalies (from each observation and model grid cell the corresponding 71‐year running monthly climatologies were subtracted), long‐term variability (>70 years) rise from the forcings and boundary conditions of the models. In addition, carrying out the assimilation on the anomaly level may result in negative absolute values of total precipitation. Franke et al. (2017) point out that EKF400v1 south from 60°S should not be used in any analysis due to errors in the model simulations. This feature is inherited in v2 because the same model simulations were involved in the assimilation. Conducting the assimilation on the full resolution of the model plus applying an improved assimilation scheme (blended covariance technique) implied larger computational burden. Hence, only a group of variables could be loaded at the same time and the climatological state vector, using randomly selected members, was generated separately for each group of variables.

## CONFLICTS OF INTEREST

The authors declare that they have no conflict of interest.

## OPEN PRACTICES

This article has earned an Open Data badge for making publicly available the digitally‐shareable data necessary to reproduce the reported results. The data is available at 10.26050/WDCC/EKF400_v2.0 Learn more about the Open Practices badges from the Center for OpenScience: https://osf.io/tvyxz/wiki.

## Supporting information

Fig S1‐S4
