# Supplementary material for: An updated global atmospheric paleo‐reanalysis covering the last 400 years
Source: Geosci Data J. 2021 May 4;9(1):89–107. doi: 10.1002/gdj3.121 (PMC9292829; doi:10.1002/gdj3.121)
Supplement: Supplementary file 1 — Fig S1‐S4 [file GDJ3-9-89-s001.pdf]

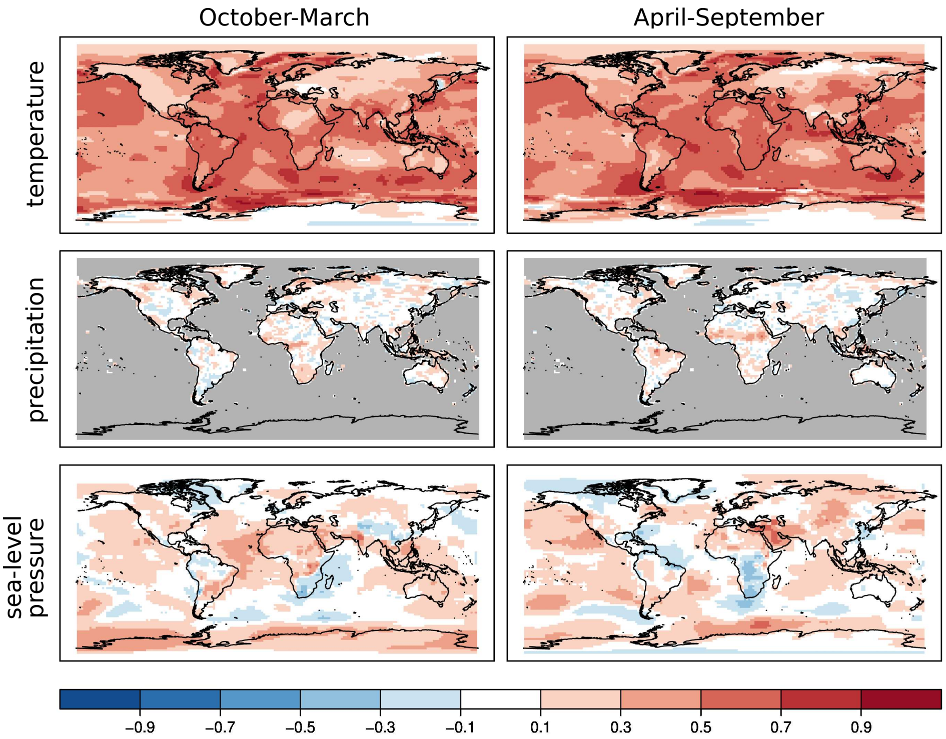

Figure S1: Spatial distribution of correlation coefficients calculated between the CCC400 ensemble mean and reference datasets in the two seasons over the 1902–2002 period. The grey shaded areas indicate the region where no reference data are available.

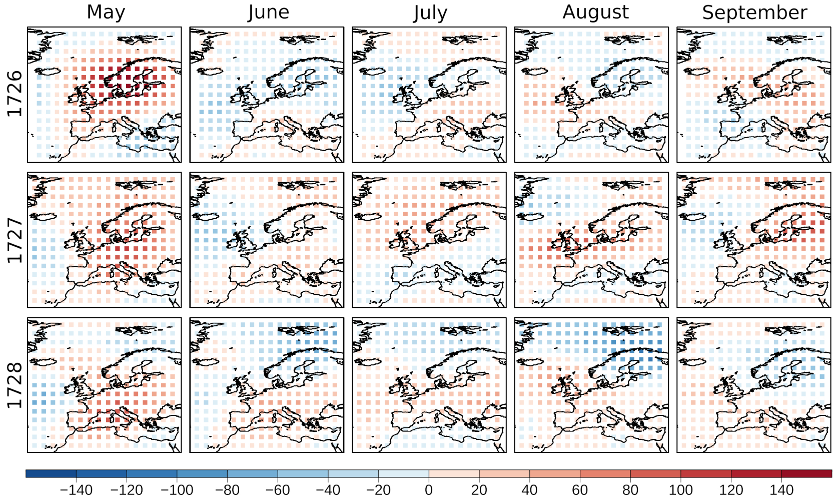

Figure S2: Monthly anomalies of 500 hPa geopotential height in EKF400v1.

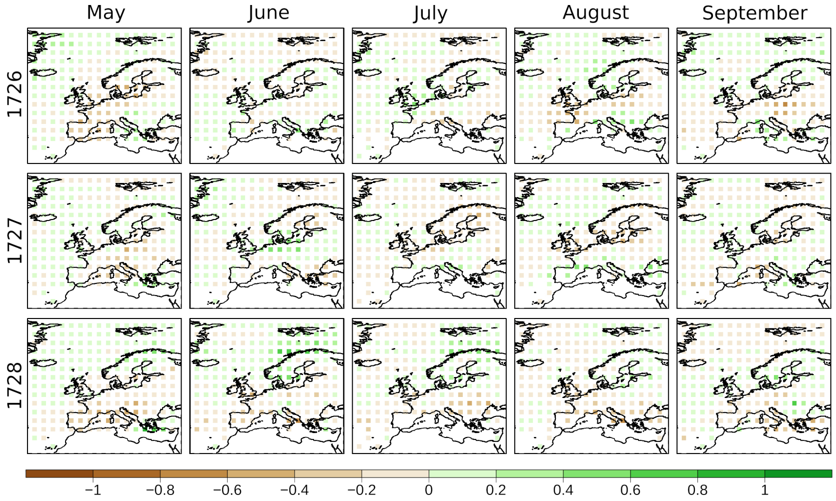

Figure S3: Monthly relative anomalies of precipitation in EKF400v1.

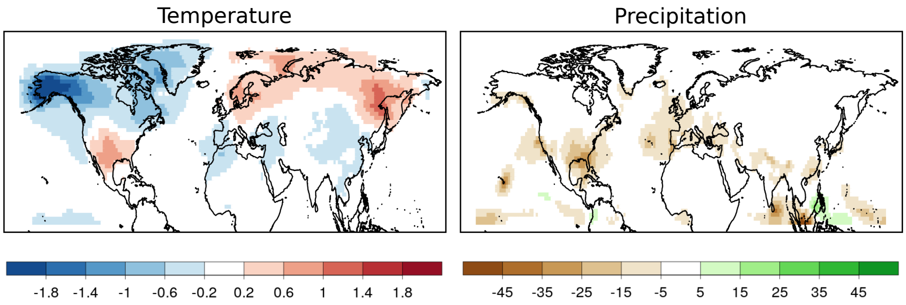

Figure S4 : Composite of temperature anomaly [K] and precipitation anomaly [mm], calculated from the La Niña years in the 20th century (1904, 1910, 1917, 1925, 1934, 1943, 1950, 1956, 1968, 1971, 1974, 1976, 1985, 1989, 2000; based on Brönnimann et al. (2007)) using EKF400v2.
